# Potential Activity, Size, and Structure of Sulfate-Reducing Microbial Communities in an Exposed, Grazed and a Sheltered, Non-Grazed Mangrove Stand at the Red Sea Coast

**DOI:** 10.3389/fmicb.2015.01478

**Published:** 2015-12-22

**Authors:** Melike Balk, Joost A. Keuskamp, Hendrikus J. Laanbroek

**Affiliations:** ^1^Department of Microbial Ecology, Netherlands Institute of EcologyWageningen, Netherlands; ^2^Faculty of Geosciences, Utrecht UniversityUtrecht, Netherlands; ^3^Ecology and Biodiversity, Department of Biology, Utrecht UniversityUtrecht, Netherlands

**Keywords:** sulfate reduction, mangroves, *Avicennia marina*, camel-grazing, DGGE fingerprinting

## Abstract

After oxygen, sulfate is the most important oxidant for the oxidation of organic matter in mangrove forest soils. As sulfate reducers are poor competitors for common electron donors, their relative success depends mostly on the surplus of carbon that is left by aerobic organisms due to oxygen depletion. We therefore hypothesized that sulfate-cycling in mangrove soils is influenced by the size of net primary production, and hence negatively affected by mangrove degradation and exploitation, as well as by carbon-exporting waves. To test this, we compared quantitative and qualitative traits of sulfate-reducing communities in two Saudi-Arabian mangrove stands near Jeddah, where co-occurring differences in camel-grazing pressure and tidal exposure led to a markedly different stand height and hence primary production. Potential sulfate reduction rates measured in anoxic flow-through reactors in the absence and presence of additional carbon sources were significantly higher in the samples from the non-grazed site. Near the surface (0–2 cm depth), numbers of *dsrB* gene copies and culturable cells also tended to be higher in the non-grazed sites, while these differences were not detected in the sub-surface (4–6 cm depth). It was concluded that sulfate-reducing microbes at the surface were indeed repressed at the low-productive site as could be expected from our hypothesis. At both sites, sulfate reduction rates as well as numbers of the *dsrB* gene copies and viable cells increased with depth suggesting repression of sulfate reduction near the surface in both irrespective of production level. Additionally, sequence analysis of DNA bands obtained from DGGE gels based on the *dsrB* gene, showed a clear difference in dominance of sulfate-reducing genera belonging to the Deltaproteobacteria and the Firmicutes between sampling sites and depths.

## Introduction

Mangrove forests, which are confined to tropical and subtropical coast lines, are known to be highly productive ecosystems with the capacity to efficiently trap suspended material from the water column (Kristensen et al., 2008). The production of above- and belowground litter as well as root exudates provide significant inputs of organic carbon to mangrove soils (Alongi, 1998). Depending on the environmental conditions, this organic carbon is oxidized by a range of aerobic and anaerobic microorganisms using a variety of electron acceptors, of which oxygen is the most preferred oxidant for thermodynamic reasons (Laanbroek, 1990). Aerobic respiration and sulfate reduction are considered to be the major pathways of mangrove-derived carbon degradation with a global share of 40–50% each (Kristensen et al., 2008). Oxygen penetration in the surface of mangrove soils is often restricted to the first 2–3 mm as has been shown for soils covered by *Avicennia marina* and *Rhizophora apiculata* (Andersen and Kristensen, 1988; Kristensen et al., 1988, 1992). Due to the presence of oxygen, sulfate reduction is often suppressed in the upper few millimeters of the soil, but underneath this oxic zone, sulfate reduction generally increases with depth (Kristensen et al., 1991, 2000, 2011).

By its ability to tolerate harsh environmental conditions such as extreme temperatures and salinity (Clough, 1993), *A. marina* dominates the mangrove vegetation along the coast of the Red Sea, where it is often found in mono-specific stands (Mandura, 1997). Many mangroves stands on the Red Sea coast have been destroyed by overgrazing by camels (Mohamed, 1984; Saifullah et al., 1989). Where grazing pressure is high, the growth of mangroves is severely impacted, resulting in stunted growth and a considerable reduction in photosynthetic biomass. As with most mangrove tree species, annual leave litter production by *A. marina* increases with tree height (Komiyama et al., 2008), so that the carbon influx to the soil is likely to be negatively affected by grazing, which in turn leads to lower microbial activity. In the Vellar–Coleroon estuarine complex at the coast of the Bay of Bengal in the state of Tamil Nadu, India, cattle-grazing resulted also in stunted stands of *A. marina*, which led to lower rates of microbial activity, especially in compacted and dry surface soils (Alongi et al., 2005). Besides the negative effects on carbon input, camel-grazing is also likely to decrease oxygen input to the sub-surface soil, as trampling deteriorates oxygen-conducting pneumatophores (Khalil, 2004). As a result, the proportion of organic matter which is reduced by sulfate reduction may increase.

Hence, at the surface of a mangrove forest soil with an intrusion of oxygen that is not affected by camel-grazing, a lower carbon input into the soil due to grazing will limit the amount of electron donors available for sulfate-reducing microorganisms since aerobic microorganisms will consume these electron donors with priority. In sub-surface layers where the intrusion of oxygen is mainly determined by the presence of pneumatophores, deterioration of these aerial roots by camel-trampling will stimulate sulfate reduction provided that sufficient carbon is available. However, a lower carbon input into the surface layer due to grazing will also lead to a lower input of electron donors in the sub-surface layers and the positive effect of deteriorated pneumatophores on sulfate reduction might by nullified by the lower carbon input. For this reason, grazing will affect the process of sulfate reduction in a non-linear and interactive way.

At South Corniche, a coastal strip of the Red Sea 20 km southwest of the city of Jeddah, camels irregularly graze *A. marina* trees growing in a narrow zone along a beach exposed to tidal currents. The height of the trees never exceeds 1 m, which is notably smaller than the trees present at more sheltered, non-grazed site about 120 km to the north. We hypothesize that the modifying effects of camel-grazing and the occurrence of waves affect the sulfate-reducing community both quantitatively and qualitatively.

To test this hypothesis, soil samples were collected at these two sites differing in their exposure to camel-grazing and tidal currents. The grazed site has a low production, as can be inferred from the stunted growth of the mangrove trees. Measured edaphic factors were rather similar between the sites, except for salinity, which was higher at the grazed site (56.0 versus 51.1 PSU). At both sites, quantitative and qualitative properties of sulfate-reducing microbial communities were determined in samples taken at the surface and sub-surface of the soils (i.e., at 0–2 cm and 4–6 cm depth). Potential sulfate reduction rates were determined in flow-through reactor (FTR) systems that allow the supply of nutrients to the soil while conserving the soil structure (Roychoudhury et al., 1998; Pallud and Van Cappellen, 2006). Numbers of viable cells were determined by a Most Probable Number (MPN) technique (Laanbroek and Pfennig, 1981). Quantitative PCR based on the functional *dsrB* gene that codes for part of the dissimilatory sulfite reductase enzyme was used for estimation of the size of the sulfate-reducing community (Müller et al., 2014). Finally, denaturing gradient gel electrophoresis (DGGE) based on the same functional gene was applied to identify the dominant sulfate-reducing microbial species at both sites and depths (Miletto et al., 2007).

## Materials and Methods

### Study Sites

Our study was conducted with soil samples from two *Avicennia marina* mangrove forests along the Red Sea coast near Jeddah, Saudi-Arabia. One site along the coast at South Corniche (N21°16′06″ and E39°07′30″) consisted of a current-exposed zone with a vegetation of *A. marina*, which was regularly grazed by camels, while the second site at the fringe of a sheltered creek (N22°19′52″ and E39°05′59″) on an island near Thuwal in front of the King Abdullah University for Science and Technology (KAUST) campus, 120 km north of South Corniche. At both sites, the trees grew in monoculture on shallow soils of weathered coral.

Whereas camels irregularly grazed the mangrove trees at South Corniche, the island near Thuwal was undisturbed. As a result of grazing, mangrove trees in South Corniche were stunted and never exceeded heights of 1 m. In the undisturbed stand near Thuwal, mangrove trees were much taller with an average height of 3.5 m (Keuskamp et al., 2013). Notwithstanding the differences in tree heights and exposure to tidal currents, the soils from both mangrove stands were poor in carbon.

At both sites, replicate samples were collected along a transect parallel to the waterfront at intermittent distances of approximately 1 m. During sampling, soil temperatures were within the range of 31 ± 2°C at both sites. Edaphic properties were also similar between both sampling sites (**Table 1**), with low total organic carbon and pH slightly above neutral. At South Corniche, salinity was somewhat higher and the soil consisted of relatively finer particles than at Thuwal.

**Table 1:** Some characteristics of the 0–10 cm layer of *Avicennia marina* forest soils collected from South Corniche and Thuwal at the Red Sea coast near Jeddah, Saudi Arabia.

Site	South Corniche	Thuwal
Soil temperature (°C)	30.1–32.5	29.2–32.7
Total organic matter (% dry solids)	0.7	0.9
pH	7.7	7.8
Salinity (PSU)	56.0	51.1
Sulfate (g/L)	1.9	1.8
Sulfur (g/L)	2.5	2.7
Mean particle size (DV50^a^)	73.0	136.0


*^a^As volume-based mean diameter.*

### Determination of Sulfate Reduction Rate

Sulfate reduction rates were determined in FTRs designed to measure rates of biogeochemical reactions, in undisturbed, water-saturated soils, and sediments (Roychoudhury et al., 1998). Each reactor contained a slice of soil within a Plexiglas ring of 2 cm thickness and 4.2 cm inside diameter, with 0.2 μm pore size PVDF (Millipore) filters and glass fiber filters (1.2 mm PALL) at each end. The reactors were closed using POM (polyoxymethylene) Delrin^®^ caps tightened with screws, whereas O-rings prevent leakage. The soils were sampled using a hand-operated shuttle corer, whose core liner consists of a stacking of reactor cells (Pallud and Van Cappellen, 2006). Undisturbed soil is thus directly collected in the stacking, with each cell corresponding to a given depth interval. The samples were taken from the soils that were close to the roots of the trees. After retrieving a core, the cell stacks were taken apart and each cell was individually closed by filters and caps. Two depth intervals were used for further analysis, i.e., from 0–2 cm and 4–6 cm depth; these depths are referred further as surface and sub-surface, respectively. All experiments were run with five replicates.

In order to purge original pore water from the soil, reactors were flushed with specific concentrations of NaCl that match the salt concentrations measured at the two sites (**Table 1**) at a constant flow rate of 1.0 ± 0.1 ml h^-1^ and at 21 ± 0.5°C for 24 h before starting the experiments. During the incubation period, the FTRs were maintained at a constant temperature of 30 ± 0.5°C by placing them in a water bath. The inflow solutions contained 4 mM sodium sulfate, defined concentrations of NaCl and no electron donor. In order to determine the effect of additional organic carbon on sulfate reduction rates, both sodium acetate (10 mM) and sodium lactate (10 mM) were supplied in the inflow solutions. At the same time the concentration of sodium sulfate was increased to 24 mM to meet the extra reductive potential in the inflow solution caused by the added acetate and lactate. Reactors supplemented with sulfate only will further be referred to as non-amended; reactors receiving both exogenous carbon and extra sulfate will be referred to as amended.

Inflow solutions and tubing were purged with argon before and during the FTR experiments to maintain anoxic conditions. Inflow solutions were introduced at a constant flow rate of 1.0 ± 0.1 ml h^-1^ using a peristaltic pump. Collection tubes pre-filled with 2 ml zinc acetate (10%) to trap sulfide, were changed at indicated fixed time intervals and then stored at –18°C prior to chemical analyses. All incubations were performed in the dark to eliminate the possibility of oxygen production through photosynthesis.

Steady state sulfate reduction rates were calculated as follows:

$$Sulfate\,\;reduction\,\;rates=(C_{in}-C_{out})\times Q/V$$

Where, *C*_in_ is the sulfate input concentration, *C*_out_ is the steady state sulfate concentration in the outflow, *Q* is the volumetric flow rate, and *V* is the volume of the soil slice in the reactor.

### Enumeration of Sulfate-Reducing Microorganisms

Densities of viable sulfate-reducing microorganisms were enumerated using a MPN assay. Soil was re-suspended in phosphate-buffered saline (PBS, per liter of milli-Q water: 8 g NaCl, 0.2 g KCl, 1.44 g Na_2_HPO_4_, 0.24 g KH_2_PO_4_; pH 7.4) in a soil to buffer ratio of 1:6. Slurries were shaken for 2 h. Homogenates were immediately used for inoculation of MPN dilution series in microtiter plates (BRAND, 8 × 12 wells of 250 μl). Tenfold serial dilutions of soil were made in a minimal salt-water medium prepared according to Widdel and Bak (1992). Na_2_SO_4_ (20 mM) was provided as the electron acceptor to select for sulfate reducers. A mix of acetate, propionate, and lactate (15 mM each) served as electron donors. The reducing agent in the MPN medium was sodium thioglycolate (0.5 mM). FeSO_4_ (0.2 mM) served as an indicator of sulfate reduction; the formation of a black FeS precipitate was indicative for sulfide formation by active sulfate reducers; black wells were counted as positive. The microtiter plates were sealed with an adhesive foil (SecurSeal^®^, Simport, Beloeil, QC, Canada) and put in anaerobic incubation bags (Anaerocult^®^ A mini, Merck, Darmstadt, Germany). In the bags, a citric acid-based catalyst was used to create an oxygen-free N_2_/CO_2_ atmosphere. The atmosphere became anoxic within 1 h after sealing the bags, as shown by an indicator strip (Anaerotest^®^, Merck, Darmstadt, Germany). Cultures were incubated at 30°C for 3 months. After counting the number of positive wells per dilution, the MPN and confidence limits were calculated using standard MPN tables (Rowe et al., 1977).

### DNA Extraction and PCR Amplification of *dsr* Gene Fragments

DNA was purified using the DNA Clean and Concentrator kit (Zymo Research, Orange, CA, USA). The quantity and quality of the extracted DNA were analyzed by spectrophotometry using an ND-1000 spectrophotometer (NanoDrop Technologies, Wilmington, DE, USA) and by agarose gel electrophoresis. The genomic DNA was stored at –20°C for further analyses.

A nested PCR approach was used to increase the sensitivity of the amplification. The first PCR amplification step yielding a ca. 1900 bp long *dsrAB* fragment was performed using the primers DRS1Fmix (equimolar mixture of DSR1F, DSR1Fa, and DSR1Fb) and DRS4Rmix (equimolar mixture of DSR4R, DSR4Ra, DSR4Rb, and DSR4Rc) as described by Loy et al. (2004). The first-step PCR mixture comprised 25 μl of 2x Premix F (Epicentre Biotechnologies, Madison, WI, USA), 25 pmol of the each primer, 1 unit of *Taq* polymerase (Invitrogen), and 50 ng of genomic DNA as template, in a total volume of 50 μl. The following PCR conditions were used: 5 min at 94°C; 30 cycles, with 1 cycle consisting of 94°C for 30 s, 55°C for 30 s, and 72°C for 90 s, and a final extension at 72°C for 10 min. Subsequently, a 350 bp fragment of the *dsrB* gene was amplified in the second step using primers DSRp2060F (Geets et al., 2006) and DSR4R. Five microliters of 1/100-diluted PCR product from the first step was used as template in the subsequent nested amplification. The second-step PCR was carried out using a touchdown protocol with an initial incubation of 5 min at 94°C, then 20 cycles of 1 min at 94°C, 1 min at the annealing temperature, and 1 min at 72°C, followed by a final incubation of 10 min at 72°C. The annealing temperature was lowered from 65°C to 55°C over the first 11 cycles, after which it was maintained for a further 14 cycles at 55°C. The yield and quality of the PCR products were examined on 1% (wt/vol) agarose gel stained with GelRed^TM^ Nucleic Acid Gel Stain.

### DGGE of *dsrB* Gene Fragments

Denaturing gradient gel electrophoresis was performed essentially as described by Muyzer et al. (1993). PCR products were separated on a 1.5 mm thick, vertical gel containing 8% (w/v) polyacrylamide (37.5:1 acrylamide:bisacrylamide) and a linear gradient of the denaturants urea and formamide, increasing from 25 to 75%. Hundred percent of denaturant is defined as 7 M urea plus 40% v/v formamide. The gels were loaded with 8–10 μl of PCR product, depending on the band intensity of the PCR product after electrophoresis on 1.5% agarose gels. Before loading, the PCR products were mixed with loading buffer (0.25 μl loading buffer per μl of PCR product). The loading buffer contained 50% Glycerol, 50 mM Tris/HCl pH 7.5, 5 mM EDTA and 0.05% bromophenol blue. Electrophoresis was performed in a buffer containing 40 mM Tris, 40 mM acetic acid, 1 mM EDTA (pH 7.6; 0.5x Tris–acetate–EDTA buffer) for 16 h at 100 V. Gels were stained for 1 h in water containing 0.5 μg ml^-1^ ethidium bromide. Images were recorded with a CCD camera mounted on the AutoChemi^TM^ Darkroom (UVP Inc. Upland, CA, USA). Bands of interest were isolated from the gel using a sterile tip and the DNA containing acrylamide fragments were incubated overnight at room temperature in sterile PCR water to allow DNA diffusion out of the polyacrylamide matrix. The solution was directly used for further amplifications. Excised bands were re-amplified using the cycling previously described (primer set DSRp2060F-GC/DSR4R, 20 cycles), and re-run on DGGE to confirm their identity and purity prior to purification and then purified using the Gel Recovery Kit (Zymoclean, Orange, CA, USA) and subjected to sequencing at Macrogen, Amsterdam, The Netherlands (http://www.macrogen.com).

### Real-Time PCR Amplification

Real-time PCR amplification for sulfate-reducing prokaryotes targeting the *dsrB* gene was performed in a total volume of 20 μl with primer pair DSRp2060F and DSR4R (Geets et al., 2006) on a Rotor-Gene 6000 thermal cycling system (Corbett Research, Sydney, NSW, Australia). Each PCR mixture contained 3 μl diluted (to 1 ng/μl) DNA template, 10 μl Absolute^TM^ QPCR SYBR Green Mix (Thermo Scientific, Epsom, UK), 0.4 μl each primer (10 μM) and 1 μl Bovine Serum Albumin (BSA; 20 mM) made using a CAS-1200 pipetting robot (Corbett Research, Sydney, NSW, Australia). The thermo profile was the following: 10 s at 95°C for initial denaturation, 45 cycles of 20 s at 95^∘o^C, 30 s at 56°C and 45 s at 72°C. The fluorescence was obtained at 84°C for each cycle. A melting curve was performed from 55°C to 99°C to confirm PCR product specificity for the reaction. Standard curves were constructed with serial dilutions of known amounts of *dsrB* gene, which were amplified with dsr4R/dsr2060F primers from pure cultures of *Desulfovibrio desulfuricans, Desulfobulbus propionicus, Desulfobacter latus*, and *Desulfococcus multivorans*. Serial dilutions covered a range of 7 orders of magnitude (10^2^–10^9^) of template copies per assay. In order to get specific products and avoid inhibition, dilution series were made of the soil DNA solution to test for inhibition and set a 100-fold dilution as the final template. The amplification efficiency ranged from 98 to 104% with *R*^2^ values greater than 0.99.

### Phylogenetic Analysis

Partial *dsrB* sequences were compared to sequences stored in the GenBank database for preliminary identification using the BLAST algorithm. Subsequently, the concatenated sequences were aligned and a phylogenetic tree was plotted using MEGA 6.06 software (Tamura et al., 2013). Partial *dsrB* nucleotide sequences determined in this study have been submitted to the EMBL database under the accession numbers KF493653 to KF493576.

### Analytical Methods

Sulfate concentrations were measured by ion chromatography using a Dionex DX120 (Water, Milford, MA, USA) with an IonPac ICE-AS6 column and Anion-ICE Micro Membrane II suppressor.

### Statistical Analyses

Data were analyzed in R 3.1.1 and were fitted to mixed linear models with treatment, sampling site and depth as fixed factors and core stack as random factor using nlme 3.1-118 (Nakagawa and Schielzeth, 2013; Pinheiro et al., 2014). Residuals were tested for heteroscedacity using Bartlett’s test and for normality using the Shapiro–Wilk test. In case of violation of either of these assumptions, this was solved through transformation of the response variable. Treatment effects were tested for significance using ANOVA with type II sum of squares using car 2.0–22 (Fox and Weisberg, 2011). Adjusted *R*^2^ for mixed models were calculated using the method of Nakagawa and Schielzeth (2013).

A principal component analysis (PCA) was performed using the PAST software package version 2.17c (Hammer et al., 2001). The same software package was also used for the determination of Shannon’s index of diversity.

## Results

### Sulfate Reduction Rates

**FIGURE 1:**
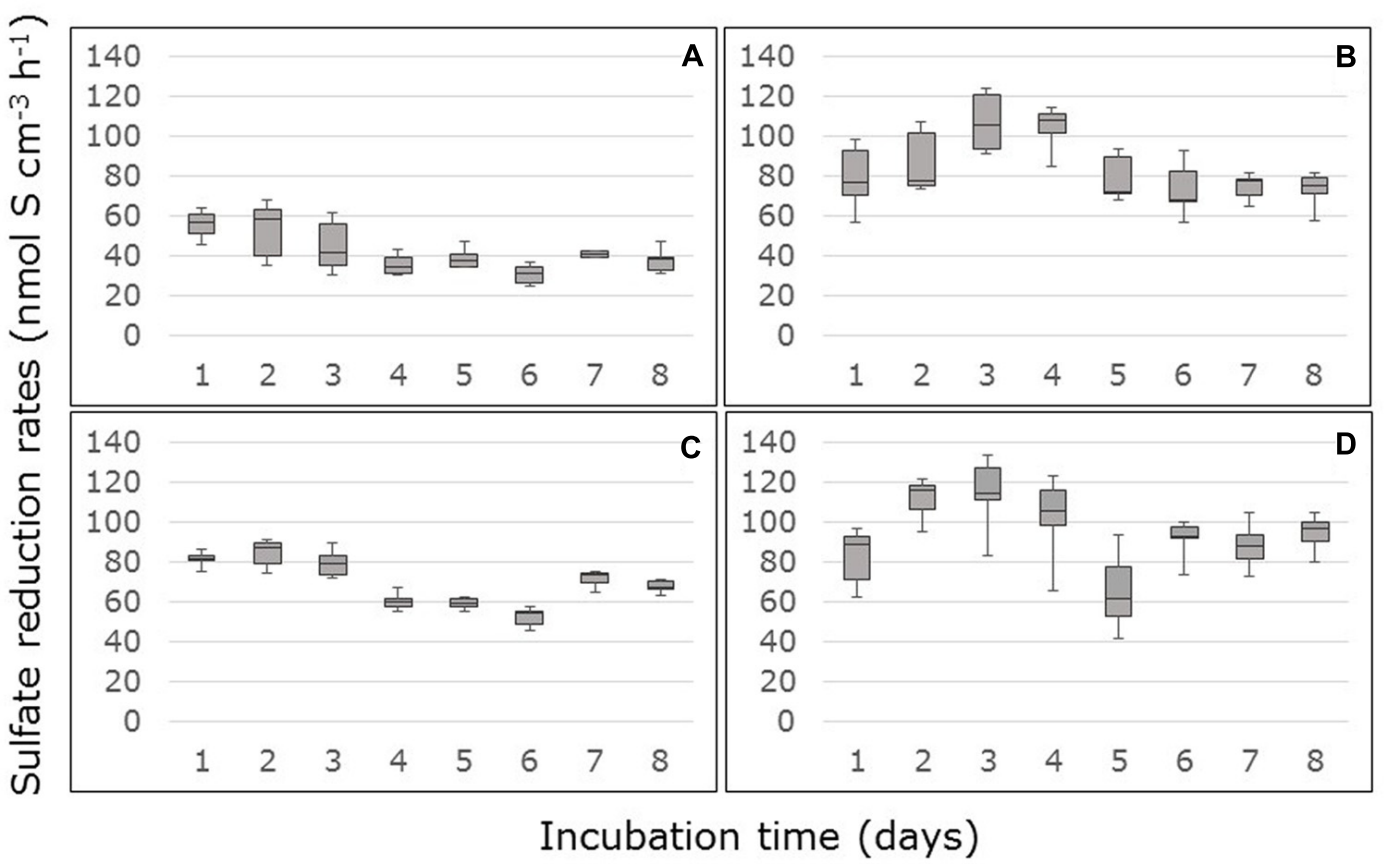
**Rates of sulfate reduction measured in flow-through reactors (FTRs) containing non-carbon-amended *Avicennia marina* mangrove soil samples collected from the surface **(A,B)** or sub-surface **(C,D)** from South Corniche **(A,C)** and Thuwal **(B,D)** at the Red Sea coast near the city of Jeddah, Saudi Arabia.** The soils were supplied with 10 mM sulfate.

Sulfate reduction rates based on differences in sulfate concentrations in the inflow and the outflow of the FTRs, declined in the first days of the measurement in the non-carbon-amended, surface samples from South Corniche and reached a steady state after 4 days (**Figure 1A**). In the non-carbon-amended samples of the sub-surface layer of this sampling site, sulfate reduction started declining after 3 days, while rates slightly increased again after 6 days (**Figure 1C**). In the non-carbon-amended samples from Thuwal, both from the surface and the sub-surface, sulfate reduction rates increased until day 3, after which they declined to a steady state (**Figures 1B,D**). The steady state sulfate reduction rates as measured from day 4 onward are summarized in **Table 2.** Steady state reduction rates of non-amended samples were significantly (*p* < 0.001, **Supplementary Table S1**) higher in cores from Thuwal than from South Corniche. The sulfate reduction rates measured in the non-amended samples from the surface layers were significantly (*p* < 0.001, **Supplementary Table S1**) lower than the rates observed in non-carbon-amended samples from the sub-surface layers. A significant, interactive effect of sampling site and depth was also observed.

**Table 2:** Average rates of steady state sulfate reduction measured in *Avicennia marina* mangrove soils from South Corniche and Thuwal at the Red Sea coast near Jeddah, Saudi Arabia.

Site	Depth layer (cm)	Steady state sulfate reduction rates (nmol cm^-3^ h^-1^)^a^	
		
		Non-amended	Carbon-amended
South Corniche	0–2	42 (9)	245 (10)
	4–6	69 (12)	369 (6)
Thuwal	0–2	84 (14)	243 (61)
	4–6	93 (5)	421 (75)


*^a^Averages were calculated using data from five replicates after sulfate concentrations in the outflow reached (near) constant values. Standard deviations are indicated in brackets.*

**FIGURE 2:**
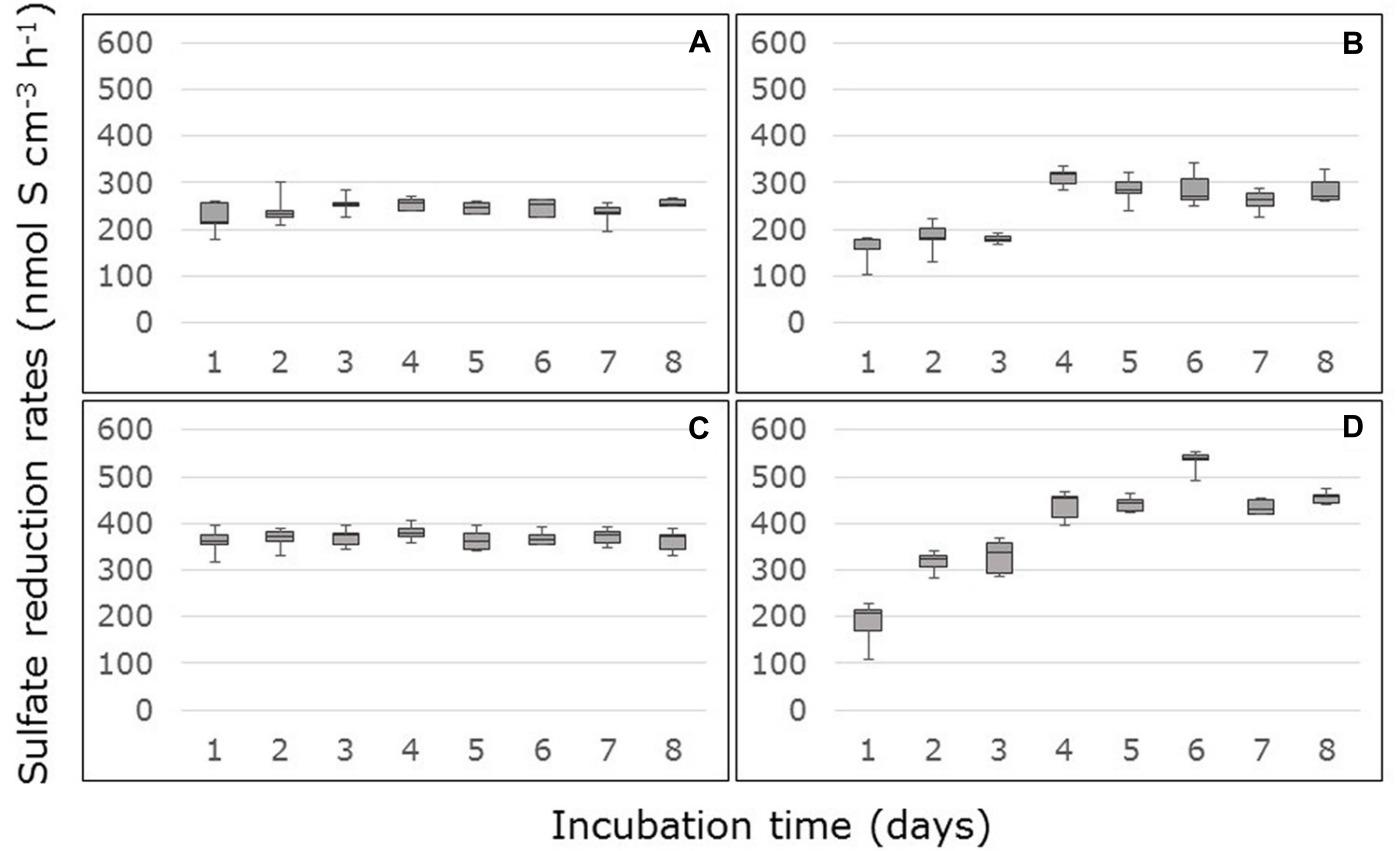
**Rates of sulfate reduction measured in FTRs containing carbon-amended *Avicennia marina* mangrove soil samples collected from the surface **(A,B)** or sub-surface **(C,D)** from South Corniche **(A,C)** and Thuwal **(B,D)** at the Red Sea coast near the city of Jeddah, Saudi Arabia.** The soils were supplied with 10 mM acetate, 10 mM lactate, and 24 mM sulfate.

Amendment with 10 mM acetate and 10 mM lactate to the medium increased the sulfate reduction rates on average by factor of 5.4 (factor range: from 3.5 for the surface samples from Thuwal to 6.8 for the surface samples from South Corniche). In the carbon-amended surface and sub-surface samples from South Corniche, sulfate reduction rates reached steady state almost directly after the start of the measurements (**Figures 2A,C**). In the carbon-amended samples from Thuwal, steady states in sulfate reduction rates were only reached after 3 days in both surface and sub-surface samples (**Figures 2B,D**). The steady state sulfate reduction rates were measured from day 4 and are summarized in **Table 2.** The sulfate reduction rates measured in the carbon-amended samples from Thuwal were significantly higher (*p* < 0.001, **Supplementary Table S2**) than the rates observed in carbon-amended samples from South Corniche. Similarly to the non-carbon-amended samples, the rates in the carbon-amended samples were significantly (*p* < 0.001, **Supplementary Table S2**) higher in the sub-surface layer than in the surface layer. A significant, interactive effect of sampling site and depth was observed again.

### Bacterial and Gene Numbers

In the samples, the copy numbers of the *dsrB* gene were in the order of 10^8^ per g soil (**Table 3**). Copy numbers of the *dsrB* gene were significantly (*p* < 0.001, **Supplementary Table S1**) affected by sampling depth with the highest numbers in the sub-surface layers. Gene copy numbers at the surface were higher at Thuwal than at South Corniche, however, this difference was not significant because of the variation in numbers between replicates.

**Table 3:** First *dsrB* gene copy numbers (qPCR) and then most probable numbers (MPN) observed in mangrove soils from South Corniche and Thuwal at the Red Sea coast near Jeddah, Saudi Arabia.

Site	Depth layer (cm)	qPCR (gene copies g^-1^ soil)^a^	MPN (cm^-3^ soil)^a^
South Corniche	0–2	2.4 × 10^8^ (0.3 × 10^8^)	2.7 × 10^5^ (1.9 × 10^5^)
	4–6	9.4 × 10^8^ (6.5 × 10^8^)	4.4 × 10^6^ (2.7 × 10^6^)
Thuwal	0–2	6.9 × 10^8^ (1.1 × 10^8^)	6.5 × 10^5^ (2.5 × 10^5)^
	4–6	7.6 × 10^8^ (0.6 × 10^8^)	3.3 × 10^6^ (2.9 × 10^6^)


*^a^Averages of 10 replicates. Standard deviations are indicated in brackets.*

The abundances of culturable sulfate-reducing microorganisms at the two mangrove stands, as determined by MPN enumerations in microtiter plates, are also presented in **Table 3.** The MPN counts showed that microorganisms capable of sulfate reduction were present at high numbers at both sites. As with *dsrB* gene copy numbers, MPN numbers were significantly (*p* < 0.001) affected by sampling depth with the highest numbers in the sub-surface layers (**Table 2**, **Supplementary Table S1**). The MPN numbers from the surface were higher at Thuwal than at South Corniche, however, this difference was not significant because of the variation in numbers between replicates.

### Community Structure Based on DGGE

**FIGURE 3:**
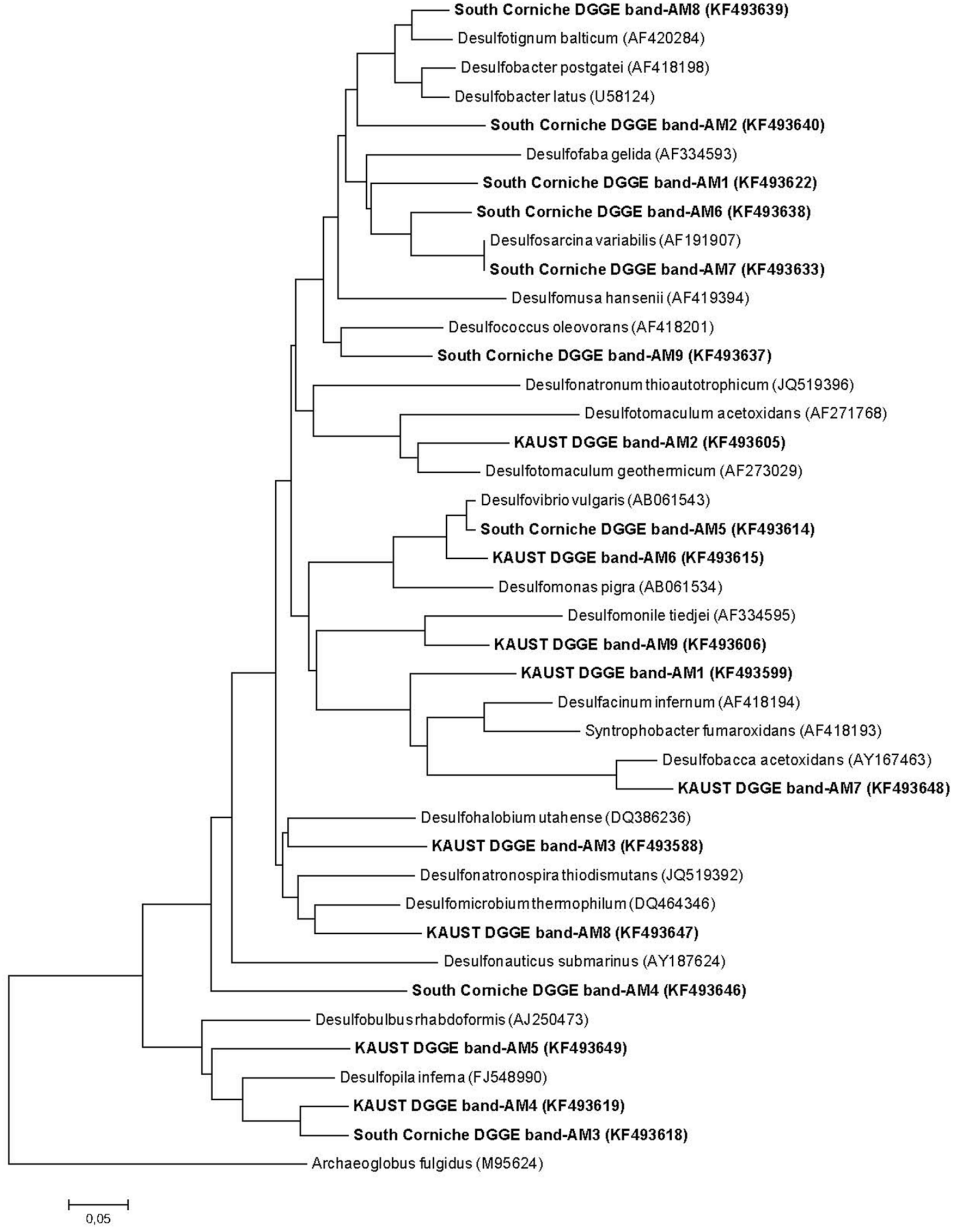
**Neighbor-joining DSR phylogenetic tree showing the affiliation of sequences obtained from DGGE-excised bands of sulfate-reducing bacteria obtained in soil samples from the South Corniche and Thuwal mangrove stands.** Phylogenetic and molecular evolutionary analyses were conducted using *MEGA* version 6.06 (Tamura et al., 2013). *Archaeoglobus fulgidus* (M95624) was used as the out-group reference.

**FIGURE 4:**
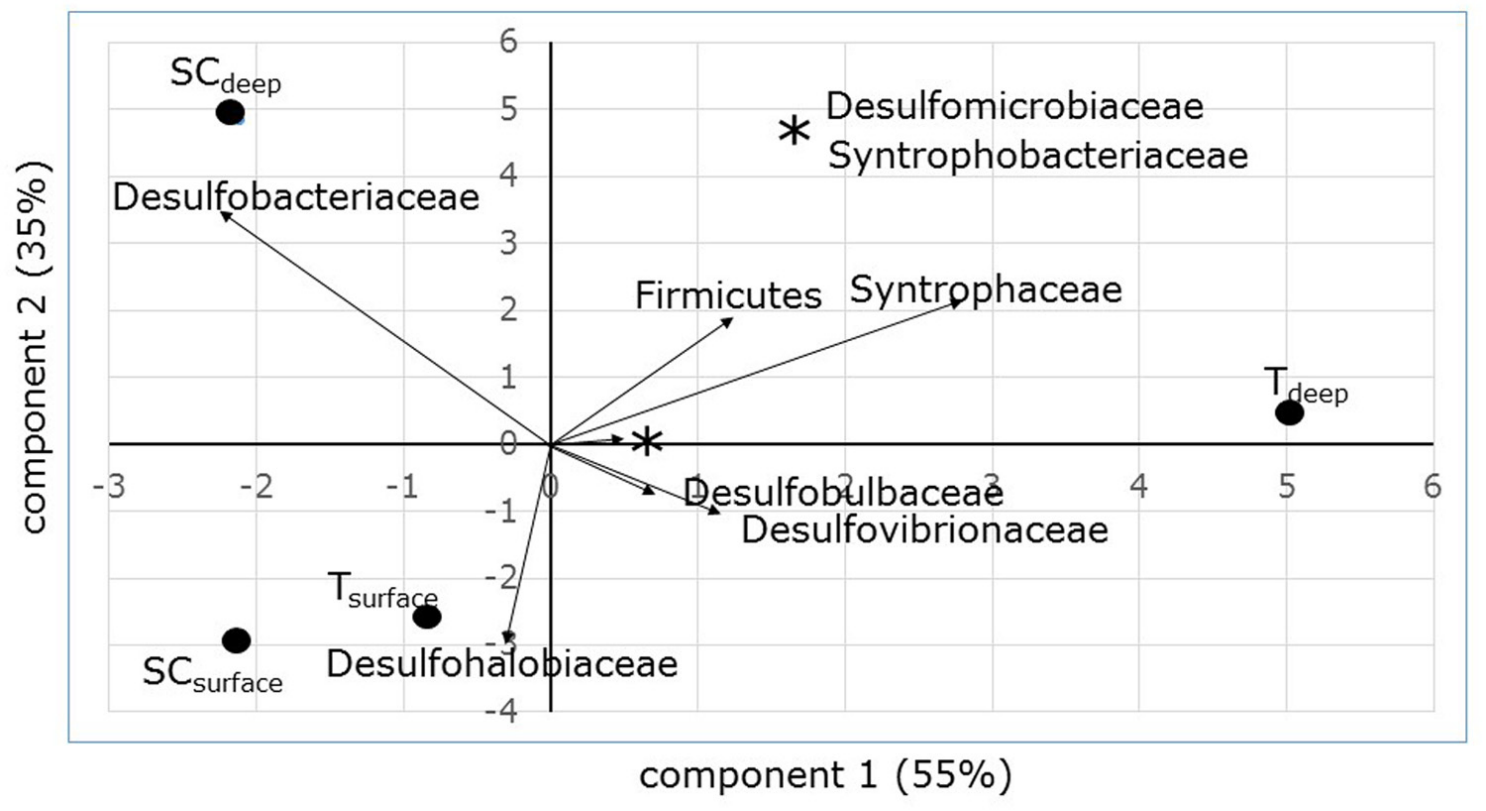
**PCA ordination biplot of sampling sites and depths based on *dsrB* sequence distribution.** Percentages at the axes explain the amount of variation explained by the axes.

Denaturing gradient gel electrophoresis based on PCR of the *dsrB* gene was used to identify differences in the dominant sulfate-reducing species between sampling sites and depths. All sequences excised from the DGGE bands were found to belong to sulfate-reducing bacteria within the phylum Proteobacteria (class Deltaproteobacteria) or from the phylum Firmicutes (**Figure 3**). The bacterial families were unevenly distributed over the different sampling units (**Table 4**). The differences in diversity between both sampling sites as shown in **Table 4** is reflected in the calculated Shannon diversity indices: 1.055 and 1.004 for the surface and sub-surface layers of South Corniche, respectively, and 1.519 and 1.708 for the surface and sub-surface layers of Thuwal, respectively. The distinct position of the sub-surface layer from Thuwal was also clear from a PCA of the retrieved sequences on the level of bacterial families (**Figure 4**). The first component, which explained 55% of the variance among the samples, separates the sub-surface layer of Thuwal from the other sampling units. The second component explaining 35% of the variance divides the surface layers from the sub-surface layers. The ordination of the sub-surface layer of Thuwal was largely determined by the presence of a relatively large number of bands belonging to the Syntrophaceae, whereas the ordinations of the surface layer of Thuwal and the sub-surface layer of South Corniche coincided with relatively large numbers of bands that fit with the Desulfohalobiaceae and the Desulfobacteraceae, respectively.

**Table 4:** Distribution of *dsrB* sequences representing sulfate-reducing bacterial families over the different sampling units.

Bacterial families	Surface		Sub-surface	
		
	South Corniche	Thuwal	South Corniche	Thuwal
Desulfobacteraceae	3	4	8	1
Desulfobulbaceae	2	2	1	3
Desulfohalobiaceae	2	5	0	1
Desulfomicrobiaceae	0	0	0	1
Desulfovibrionaceae	1	2	0	3
Syntrophaceae	0	2	3	7
Syntrophobacteriaceae	0	0	0	1
Firmicutes	0	2	3	4


In the DGGE gels, a total of 15 different bands were observed, of which 11 bands could be assigned to genera with known physiological abilities (**Table 5**). Most of the genera were exclusively found at one of the sampling sites. Only the genus *Desulfopila*, which belongs to the Desulfobulbaceae, and an unknown genus of the Desulfovibrionaceae were detected at both sites. In contrast to the almost exclusive distribution of the genera over the sampling sites, most of the genera were observed at both the surface and the sub-surface layers. Exceptions were the genera *Desulfobacter*, *Desulfosarcina* (both Desulfobacteraceae), and *Desulfonauticus* (Desulfohalobiaceae) that were only detected at the surface layer of South Corniche. The genus *Desulfobacterium* (Desulfobacteraceae) and the genera *Desulfobacca* and *Desulfomonile* (both Syntrophaceae) were only found in the sub-surface layers of South Corniche and Thuwal, respectively.

**Table 5:** Distribution and potential electron donor range of sulfate-reducing, bacterial genera detected in *Avicennia marina* forest soils along the Red Sea Coast of Saudi Arabia.

Family	Genus	Sampling site and depth				Potential electron donors based on literature data	Reference
				
		South Corniche		Thuwal			
					
		surface	sub-surface	surface	sub-surface		
Desulfobacteraceae	*Desulfobacter*	+				Acetate and ethanol	Widdel, 1987
	*Desulfobacterium*		+			Methanol, **glutarate, glutamate, phenol, aniline, nicotinate, indole**	Cravo-Laureau et al., 2004
	*Desulfosarcina*	+				H_2_, fatty acids, ethanol, **phenyl-substituted organic acids**	Widdel, 1980; Rabus et al., 2006
	Unknown	+	+				
	Unknown			+	+		
Desulfobulbaceae	*Desulfobulbus*			+	+	H_2_, ethanol, propionate, lactate	Widdel and Pfennig, 1982; Rabus et al., 2006
	*Desulfopila*	+	+	+		Lactate, alcohols	Suzuki et al., 2007
Desulfohalobiaceae	*Desulfonauticus*	+	+			H_2_	Audiffrin et al., 2003
Desulfovibrionaceae	*Desulfovibrio*			+		H_2_, methanol, ethanol, lactate, glycerol, **glycine, alanine, choline, furfural**	Rabus et al., 2006
	unknown	+	+		+		
Syntrophaceae	*Desulfobacca*				+	Acetate and ethanol	Oude Elferink et al., 1999; Rabus et al., 2006
	*Desulfomonile*				+	H_2_, **phenyl-substituted organic acids, 3- or 4-anisate**	Rabus et al., 2006
	unknown			+	+		
Syntrophobacteraceae	*Syntrophobacter*			+	+	Propionate	Boone and Bryant, 1980; Harmsen et al., 1993
Firmicutes	*Desulfotomaculum*			+	+	Methanol, ethanol, alanine	Widdel and Pfennig, 1977


*Presence of genera is based presence on DGGE gels. Bold: Relatively poorly oxidizable electron donors.*

## Discussion

At least for the surface layer, the results support our hypothesis that factors such as camel-grazing and waves affect the sulfate-reducing microbial community. Independent from the absence or presence of amended carbon, the steady state sulfate reduction rates determined in the FTRs were significantly lower in soil samples from the exposed and grazed site at South Corniche than in samples from the sheltered and non-grazed site at Thuwal. Assuming that these rates reflected the size of the sulfate-reducing community that is active *in situ*, it can be concluded that the environmental conditions at the sheltered and grazed site had a negative effect on the size of sulfate reduction in the sampled soils. We infer that this must result from a lower availability of electron donors at this site, as the alternative, an increase in oxygen flux through grazing seems highly unlikely. In non-carbon-amended reactors, steady state sulfate reduction rates were significantly larger in the sub-surface layers than in the surface layers, which meant that the amount of carbon available for sulfate reduction was larger in the deeper anoxic layers. This might have been due to the absence of competition for carbon with aerobic microorganisms. Not only steady state sulfate reduction rates, but also the numbers of *dsrB* gene copies and viable sulfate-reducing cells increased significantly with depth. However, a significant effect of sampling site on these numbers was not observed. Whereas numbers in the surface samples were higher at the sheltered, non-grazed site, numbers in the sub-surface samples were higher at the exposed, grazed site. Although rates and numbers followed generally the same trends between grazed and non-grazed and between surface and sub-surface, lower rates but higher numbers were observed in the sub-surface layer of the grazed site when compared to the sub-surface layer of the non-grazed site. This demonstrates that rates and numbers of *dsrB* gene copies and viable cells are not necessarily coupled.

### Stimulating or Inhibiting Effects

Howarth et al. (1995) and Sherman et al. (1998) showed that rates of sulfate reduction not only depends on organic matter input, but also on physical processes that affect mixing and irrigation of surface sediments. The sheltered site at Thuwal might experience less mixing than the tidal currents-exposed site at South Corniche. Our observation that the development of sulfate reduction rates during the first days of incubation was markedly different between the sites may be related to the scale of mixing at both sites. While the rates in the samples collected at South Corniche were already high from the start of the incubation on, the samples from Thuwal showed an initial phase of three or more days with increasing sulfate reduction rates before they stabilized. This phase cannot be attributed to electron donor availability, as a similar lag phase was also observed when external carbon was provided. Rather, the initial lag phase points to induction of activity or growth of a partly inactive community of sulfate-reducing microorganisms. Flushing of soil cores with the salt solution containing sulfate with or without electron donors can have several effects. It may bring microorganisms and substrate together, which can lead to increasing sulfate-reducing activities. The fact that the increase in sulfate reduction rates occurred more or less to the same extent in the absence and in the presence of additional carbon makes this explanation less likely for the observed increase in activity in the Thuwal soils. Yet, the release of a growth- or activity-stimulating factor other than the carbon and electron donor source upon flushing can still explain the observed phenomenon of the initial increase in activities in the Thuwal soil. Conversely, a growth- or activity-suppressing factor may be washed out from the reactors upon flooding with mineral medium. An elimination of oxygen in the soils is thereby less probable as the initial increase also occurs in the permanently anoxic sub-surface layer. A reduction of sulfide is also not very likely, unless the initial sulfide concentration is equal between layers, but different between the Thuwal and South Cornice sites. This possibility cannot be excluded as sulfide concentrations have not been measured in either site. Finally, inhibitory compounds such as tannins (Scalbert, 1991; Maie et al., 2008) may inhibit sulfate reduction in the absence of a good flushing. As significant amounts of tannins are leached from decomposing *Avicennia* leaf material (Hai and Yakupitiyage, 2005), the higher plant biomass, the absence of grazing by camels, and the lower export of carbon due to a diminished tide effect, may have led to higher tannin concentrations at the Thuwal sites.

### Sulfate Reduction Rates in Perspective

Depth-integrated sulfate reduction rates in different *A. marina* forests ranged from 2 to 319 mMol S m^-2^ d^-1^ representing 20–85% of total carbon mineralization (Kristensen et al., 1992; Alongi et al., 2000, 2005). An observation of lower sulfate reduction rates in cattle-grazed mangrove forest compared to more pristine forest in the same region (Alongi et al., 2005) confirms our observation of lower rates in the camel-grazed *A. marina* stand at the Red Sea coast. The depth of soil layers with the highest sulfate reduction rate depended on local conditions, and may reach 600 nmol S cm^-3^ d^-1^ (Kristensen et al., 1992; Alongi et al., 2000, 2005; Kristensen and Alongi, 2006). Exceptionally high rates of 6000 nmol S cm^-3^ d^-1^ have been observed during the wet monsoon season (Alongi et al., 2005). A sulfate reduction rate of 600 nmol S cm^-3^ d^-1^ is approximately three times lower than the steady state rates measured in our FTRs. However, it should be kept in mind that our rate measurements were only meant for comparison between sampling depths and sites, not for determining *in situ* rates. Although the reactors contained structurally undisturbed soils, the continuous flow of sulfate-rich medium through the soils will have a positive effect on the electron donor availability in these naturally carbon-limited soils as was observed with the increase in activity when easily degradable carbon is added in the form of acetate and lactate.

### Occurrence of Sulfate Reducing Genera in Mangrove Soils

Sulfate-reducing community compositions in mangrove forest soils have rarely been studied. From the genera that emerged in our analyses, *Desulfovibrio*, *Desulfotomaculum*, *Desulfosarcina*, and *Desulfococcus* species had previously been isolated from mangroves in Goa, India, on media containing lactate, acetate, propionate, butyrate, or benzoate (Bharathi et al., 1991). Varon-Lopez et al. (2014) analyzed the composition of sulfate-reducing communities in mangrove forests in São Paulo State, Brazil, that were mainly composed of *Avicennia schaueriana*, *Laguncularia racemosa*, and *Rhizophora mangle* (Andreote et al., 2012) and that were polluted to different degrees. Pollution had an effect on the composition of the communities that comprised two major taxonomic groups affiliated with Deltaproteobacteria; one related to the order Desulfobacterales and another to the order Desulfovibrionales. Since mixed samples had been collected over a depth of 30 cm, this study could not disclose an effect of depth on the distribution of taxonomic groups. Mixing of the soil over such a depth may also explain why genes copy numbers determined by Varon-Lopez et al. (2014) were approximately three orders of magnitude lower than gene numbers at the Red Sea coast that were determined in the upper 6 cm of the soil.

### Distribution of Potential Physiological Abilities

It might be expected that organic carbon recalcitrance increases with soil depth, due to a higher average age and larger fraction of root material, which is highly refractory in comparison to leaf litter (Middleton and McKee, 2001). Hence, sulfate-reducing microorganisms that are able to use recalcitrant carbon sources as electron acceptors may preferentially be found in the sub-surface layers. Of the four detected genera, which are capable of decomposing more complex carbon compounds, two were exclusively found in sub-surface layers [i.e., *Desulfobacterium* (Desulfobacteraceae) and *Desulfomonile* (Syntrophaceae)], and two were detected exclusively in surface layers [i.e., *Desulfosarcina* (Desulfobacteraceae) and *Desulfovibrio* (Desulfovibrionaceae)]. Hence, no clear pattern emerges from the distribution of physiological abilities over the sampling depths.

A similar non-specific distribution is found with regard to the ability to use H_2_ as electron donor for the reduction of sulfate. A number of these genera (see **Table 4**) can use H_2_ as electron donor for the reduction of sulfate and could therefore function as hydrogen-consuming partners in fermentative microbial communities (Muyzer and Stams, 2008). However, establishing the occurrence of interspecies H_2_ transfer in mangrove soils requires further research since the distribution of genera with the ability to use H_2_ as electron donor for sulfate reduction does not seem to be determined by sampling site or depth.

### Potential *dsrB* Gene Bias

All sequences derived from the mangrove soil samples were either affiliated with the phylum Proteobacteria (class Deltaproteobacteria) or with the phylum Firmicutes. Sequences affiliated with sulfate-reducing microorganisms from other bacterial phyla such as Thermodesulfobacteria and Nitrospirae (with *Thermodesulfovibrio* species) or from the archaeal phyla Euryarchaeota (with *Archaeoglobus fulgidus*) and Crenarchaeota (with *Caldivirga maguilingensis*) were not detected. This could mean that sulfate-reducing bacteria other than Proteobacteria and Firmicutes are not present in mangrove soils, or that the primers used for detecting the *dsrB* gene have a bias toward these phyla. Generally, studies applying the *dsrB* gene in a range of ecosystems detected only sulfate-reducing bacteria that are affiliated with the phyla Proteobacteria or Firmicutes (Foti et al., 2007; Kjeldsen et al., 2007; Miletto et al., 2010; Pester et al., 2010, 2012; Varon-Lopez et al., 2014), except for studies related to active deep-sea hydrothermal vent chimney structures (Nakagawa et al., 2004) and non-sulfidic, mobile tropical deltaic muds (Madrid et al., 2006) that detected sequences of thermophilic, sulfate-reducing prokaryotes. The absence of such more extreme conditions may be the reason why sulfate-reducing bacteria of the phylum Thermodesulfobacteria and the genus *Thermodesulfovibrio* and sulfate-reducing archaea of the genera *Archaeoglobus* and *Caldivirga* have not been found in mangrove soils.

Since our study was primarily meant to detect quantitative differences in sulfate reduction between grazed and non-grazed mangrove sites, we did not analyze the community composition of the sulfate-reducing microorganisms extensively by extended clone or amplicon libraries, but scanned the communities by DGGE coupled to PCR that was based on the *dsrB* gene. Although a DGGE analysis will not expose the total diversity of sulfate-reducing microbes as may be expected from the other molecular analyses mentioned, it will give insight in possible differences in dominant genera.

## Conclusion

Lower levels of steady state sulfate reduction rates, lower numbers of the sulfate reduction-specific *dsrB* genes and lower quantities of culturable sulfate-reducing microorganisms in the surface layer from South Corniche compared to the upper layer from Thuwal suggested that sulfate reduction is less important at the first few millimeters of the grazed site. Such a repression of sulfate reduction may have different causes. Grazing of mangrove by camels leading to stunted trees with consequently repressed primary production will lead to a lower input of carbon into the soil at South Corniche. With an unchanged supply of oxygen and a priority use of carbon by aerobic microorganisms, less carbon will be available to the sulfate-reducing community. However, other potential mechanisms affecting sulfate reduction rates differentially at the grazed and non-grazed sites should also be considered. Export of carbon by tidal currents will be more intense at the exposed site at South Corniche, and hence could intensify a potential carbon shortage for the sulfate-reducing microorganisms at this site. In addition, the activity of sulfate-reducing microorganisms at the exposed site might be repressed by more intensive mixing due to tidal currents leading to larger oxygen availabilities. Deterioration of pneumatophores at the grazed sites may have decreased oxygen availability in the sub-surface layer leading to more electrons and carbon available for the sulfate-reducing community. Although numbers of genes and viable cells were larger in the sub-surface layer of the grazed site compared to this layer of the non-grazed site, steady state sulfate reduction rates were lower. Hence, we are not able to draw conclusions on the effect of deteriorated pneumatophores on the characteristics of the sulfate-reducing community.

On the other hand, a larger input of carbon from local primary production into the soil and a diminished export of produced carbon at the non-grazed and sheltered site at Thuwal may lead to accumulation of compounds such as tannins that can inhibit the activity of carbon-degrading microbes. Longer lag-times in sulfate reduction as observed after starting the FTRs with soils from Thuwal may be the result of leaching of inhibiting compounds such as tannins from the reactors.

In summary, the observed effects cannot with certainty be ascribed to the absence or presence of camel-grazing, since the sites do not only differ by this characteristic, but also by other traits such as the degree of exposure to tidal currents. All the characteristics at the grazed site, i.e., low primary production due to camel-grazing and the high exposure to tidal currents, will all lead to a lower input of carbon into the soil. This lower input of carbon and electrons will repress the size of sulfate reduction in the presence of a more superior electron-consuming aerobic community. This repression of sulfate reduction is reflected by trends of lower rates and numbers at the exposed, grazed site compared to the sheltered, non-grazed site.

## Author Contributions

MB contributed to the planning, sample collection, conducting the experiments, analysis of the results, and to the writing of the manuscript.

JK contributed to the sample collection, analysis of the results, and to the writing of the manuscript.

HL contributed to the planning, sample collection, analysis of the results, and to the writing of the manuscript.

## Conflict of Interest Statement

The authors declare that the research was conducted in the absence of any commercial or financial relationships that could be construed as a potential conflict of interest.
