# Supplementary material for: Potential Activity, Size, and Structure of Sulfate-Reducing Microbial Communities in an Exposed, Grazed and a Sheltered, Non-Grazed Mangrove Stand at the Red Sea Coast
Source: Front Microbiol. 2015 Dec 22;6:1478. doi: 10.3389/fmicb.2015.01478 (PMC4686736; doi:10.3389/fmicb.2015.01478)
Supplement: Supplementary file 1 [file Table_1.DOCX]

**Supplementary Table 1**⏐ ANOVA table for steady state sulfate reduction rates measured in sulfate-saturated, non-carbon-amended and carbon-amended flow-through reactors filled with surface (0 – 2 cm deep) layers and sub-surface (4 – 6 cm deep) layers from stands of *Avicennia marina* collected from South Corniche and Thuwal, Saudi Arabia.

| **Dependent variables** | **Independent variable** | **Chi^2^** | **Df** | **p (>Chi^2^)** | |
| --- | --- | --- | --- | --- | --- |
| Non-carbon-amended sulfate reduction rate | Depth | 46.864 | 1 | 7.608e-12 | *** |
|  | Location | 218.750 | 1 | <2.2e-16 | *** |
|  | Depth : Location | 14.464 | 1 | 0.0001428 | *** |
| Carbon-amended sulfate reduction rate | Depth | 628.173 | 1 | <2.2e-16 | *** |
|  | Location | 110.282 | 1 | <2.2e-16 | *** |
|  | Depth : Location | 19.353 | 1 | 1.087e-05 | *** |

Significance codes: *** 0.001, ** 0.01, * 0.05
