# Supplementary material for: Potential Activity, Size, and Structure of Sulfate-Reducing Microbial Communities in an Exposed, Grazed and a Sheltered, Non-Grazed Mangrove Stand at the Red Sea Coast
Source: Front Microbiol. 2015 Dec 22;6:1478. doi: 10.3389/fmicb.2015.01478 (PMC4686736; doi:10.3389/fmicb.2015.01478)
Supplement: Supplementary file 2 [file Table_2.DOCX]

**Supplementary Table 2**⏐ ANOVA table for numbers of *dsrB* gene copies and viable sulfate-reducing cells encountered in mangrove forest soils sampled at the surface (0 – 2 cm deep) or sub-surface (4 – 6 cm deep) layers in stands of *Avicennia marina* located at South Corniche and Thuwal, Saudi Arabia.

| **Dependent variables** | **Independent variable** | **Chi^2^** | **Df** | **p (>Chi^2^)** | |
| --- | --- | --- | --- | --- | --- |
| *dsrB* gene copy numbers | Depth | 11.595 | 1 | 0.0006614 | *** |
|  | Location | 0.4212 | 1 | 0.5163168 |  |
|  | Depth : Location | 2.1723 | 1 | 0.1405127 |  |
| Viable cell numbers | Depth | 71.416 | 1 | <2.2e-16 | *** |
|  | Location | 1.273 | 1 | 0.259210 |  |
|  | Depth : Location | 10.260 | 1 | 0.001359 | ** |

Significance codes: *** 0.001, ** 0.01, * 0.05
